# Hidden Places for Foodborne Bacterial Pathogens and Novel Approaches to Control Biofilms in the Meat Industry

**DOI:** 10.3390/foods13243994

**Published:** 2024-12-11

**Authors:** Virgínia Farias Alves, Leonardo Ereno Tadielo, Ana Carolina Moreira da Silva Pires, Marita Gimenez Pereira, Luciano dos Santos Bersot, Elaine Cristina Pereira De Martinis

**Affiliations:** 1 Faculdade de Farmácia, Universidade Federal de Goiás, Goiânia 74605-170, Brazil; virginia_alves@ufg.br; 2 Department of Animal Production and Food, State University of Santa Catarina, Lages 88040-900, Brazil; leonardoerenotadielo@gmail.com; 3 Ribeirão Preto School of Pharmaceutical Sciences, University of São Paulo, São Paulo 05508-220, Brazil; acaroliinapires@gmail.com (A.C.M.d.S.P.); maritagimenez@hotmail.com (M.G.P.); 4 Department of Veterinary Sciences, Federal University of Paraná, Palotina 85953-128, Brazil; lucianobersot@ufpr.br

**Keywords:** multispecies biofilms, foodborne pathogens, biofilm microbiota, meats, biofilm control, meat processing

## Abstract

Biofilms are of great concern for the meat industry because, despite the implementation of control plans, they remain important hotspots of contamination by foodborne pathogens, highlighting the need to better understand the ecology of these microecosystems. The objective of this paper was to critically survey the recent scientific literature on microbial biofilms of importance for meat safety and quality, also pointing out the most promising methods to combat them. For this, the databases PubMed, Scopus, Science Direct, Web of Science, and Google Scholar were surveyed in a 10-year time frame (but preferably papers less than 5 years old) using selected keywords relevant for the microbiology of meats, especially considering bacteria that are tolerant to cleaning and sanitization processes. The literature findings showed that massive DNA sequencing has deeply impacted the knowledge on the species that co-habit biofilms with important foodborne pathogens (*Listeria monocytogenes*, *Salmonella*, pathogenic *Escherichia coli*, and *Staphylococcus aureus*). It is likely that recalcitrant commensal and/or spoilage microbiota somehow protect the more fastidious organisms from harsh conditions, in addition to harboring antimicrobial resistance genes. Among the members of background microbiota, *Pseudomonas*, *Acinetobacter*, and *Enterobacteriales* have been commonly found on food contact and non-food contact surfaces in meat processing plants, in addition to less common genera, such as *Psychrobacter*, *Enhydrobacter*, *Brevundimonas*, and *Rothia*, among others. It has been hypothesized that these rare taxa may represent a primary layer in microbial biofilms, offering better conditions for the adhesion of otherwise poor biofilm formers, especially considering their tolerance to cold conditions and sanitizers. Taking into consideration these findings, it is not only important to target the foodborne pathogens per se in cleaning and disinfection plans but the use of multiple hurdles is also recommended to dismantle the recalcitrant structures of biofilms. In this sense, the last part of this manuscript presents an updated overview of the antibiofilm methods available, with an emphasis on eco-friendly approaches.

## 1. Introduction

Biofilms, with their ability to adhere, persist, and resist sanitization, pose significant food safety risks in the meat industry, where contamination by pathogenic microorganisms can lead to serious public health issues and economic losses. Industrial surfaces, such as stainless steel, polypropylene, polystyrene, and rubber, can offer sites for biofilm formation, especially considering that they are prone to cracking and biofouling, caused by regular operation or, even worse, by overuse. Moreover, the easy availability of nutrients, along with the presence of conditioning films (consisting of water lamina, fats, and proteins), combined with the difficulties in cleaning and sanitization procedures, serve as additional environmental factors that can favor microbial growth. Once established in industrial settings, biofilms are very challenging to eradicate, since they are complex structures not completely understood from the microbial ecology point of view. A better understanding of the relationships among microorganisms in biofilms is essential to propose assertive methods to control microbial contamination.

According to the classical definition, biofilms are communities of microorganisms embedded within a self-produced (or environmentally based) Extracellular Polymeric Matrix (EPS), which facilitates auto-aggregation, adhesion to biotic and abiotic surfaces, or adhesion to interfaces [1]. Microbial cells in biofilms can be protected from shear forces and stresses, such as desiccation, reaction from the immune system, protozoa ingestion, and the action of toxic compounds [2].

These microbial communities are usually regarded as hotspots for contamination in medical settings or food industries, but interestingly, they may also have biotechnological applications [3]. In fact, the ability of biofilms to withstand adverse conditions make them useful for the production of enzymes, biopharmaceuticals, water treatment, and even for the potential generation of electricity in microbial fuel cells [2].

**Figure 1:**
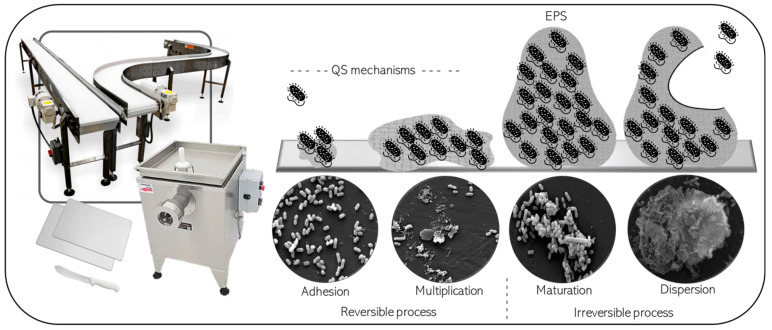
Stages of biofilm formation on industrial surfaces used for food handling. Planktonic cells approach food contact and or non-food contact surfaces and, depending on physico-chemical factors, can reversibly adhere and multiply. These initial steps may be guided by cell-to-cell communication mediated by Quorum-Sensing (QS) signaling molecules. Next, the sessile microorganisms form a matrix composed of Extracellular Polymeric Substances (EPSs), which characterize a mature biofilm, and the cells adhere irreversibly. Finally, mature biofilms can shed cells with the ability to colonize other sites in a process called dispersion, which can be mediated by several mechanisms. Source: the authors, based on [1].

Biofilms have been studied for decades and the steps of their formation and dispersal have been proposed, although many questions are open about the mechanisms involved in these processes [3,4,5]. Biofilm formation starts with the reversible adhesion of bacterial cells to biotic or abiotic surfaces or interfaces, followed by irreversible adhesion, growth, and the formation of microcolonies. Initially, cells existing in a planktonic state transition to a multicellular lifestyle, orchestrated by environmental and intrinsic signals at various regulatory levels, leading to the activation of biofilm-related effectors [4,5]. Figure 1 illustrates the steps proposed for biofilm formation, in the context of the meat processing industry.

It has been shown that the lifestyle of microorganisms in biofilms involves the fine-tuned regulation of different factors, which contribute to the performance of collective functions, many times resembling the behavior of differentiated cells in multicellular organisms [6]. Sessile microorganisms can adapt to microenvironments within biofilms, withstanding gradients of oxygen, pH, nutrient availability, and the presence of signaling molecules, such as nitric oxide (involved in biofilm dispersal) and Quorum-Sensing (QS) regulators (which are autoinducer molecules produced depending on the population density, with the ability to influence gene expression) [5,7].

**Figure 2:**
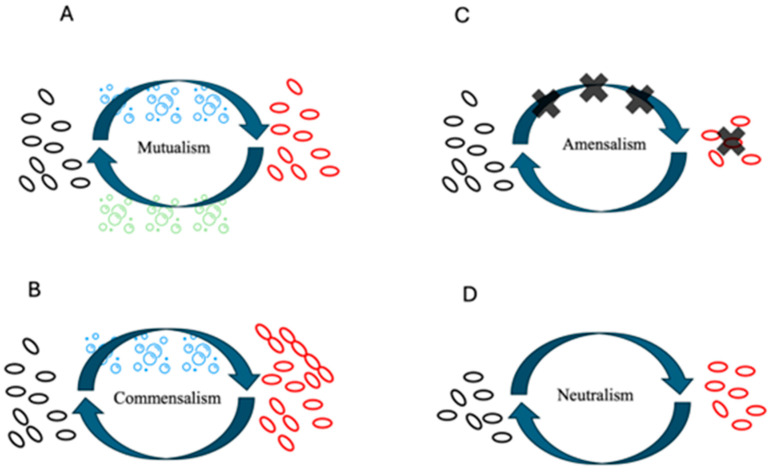
Intercommunication between two microbial populations (symbolized by black and red elongated circles): (**A**) Mutualism both organisms benefit from the association. E.g.: cross-feeding, with reciprocal benefits for both organisms by exchanging metabolites or by removing one another’s inhibitory substances (symbolized by blue and green bubbles). (**B**) Commensalism one organism is favoured, with no harm to the other. E.g.: one organism scavenges products released by the other (symbolized by blue bubbles), with no prejudice for the latter. (**C**) Amensalism one organism thrives at the expense of its partner (symbolized by black X). E.g.: production of antimicrobial compounds and competition by nutrients. (**D**) Neutralism two organisms live in the same microenvironment but there is no interaction between them. Source: the authors, based on Islam et al. [10].

Microorganisms were primarily considered to live freely, but biofilms somehow indicate their ability to “socialise” and take advantage of life in their community [8]. This is reinforced by the fact that biofilms are the natural mode of growth of up to 80% of microorganisms living on our planet [4]. Life in biofilms can be seen as a symbiosis (which in Greek means “living together”), since there is a prolonged association between two or more organisms. Figure 2 briefly illustrates the main possibilities of the interactions in biofilms, but there are no clear-cut lines in symbiotic associations, which are complex and poorly understood, especially in the microbial world [9,10,11].

According to Penesyan et al. [6], biofilms can be studied in three different complementary ways: (1) as a microbial lifestyle (a collection of individual cells, in a single-cell-centric view); (2) as an entity with characteristics of a multicellular organism (biofilm-centric view); and (3) as a diversity incubator for microbes. It is very important to consider this knowledge in order to “think outside the box” and try to envision novel ways to combat biofilms, since, so far, microorganisms have been able to overcome all the obstacles we have posed to eliminate them in the food processing industry.

From the traditional single-cell point of view, biofilms are considered solely as collections of individual microbial cells. However, in the study of biofilms as heterogenous multicellular organisms, specific processes may provide central targets for controlling microbial growth and persistence, including QS-coordinated cell behavior, the presence of gradients, and specific microenvironments inhabited by specialized cells, in addition to channels for nutrient and gas exchange [6]. With regard to QS, for example, one antibiofilm strategy includes the inhibition of enzymes that synthesize cyclic di-GMP, which is a widely conserved secondary messenger signal for biofilm formation [5,6].

The degradation of the EPS matrix is also a possibility for collapsing biofilms and promoting cell dispersal, in addition to breaking transport channels important for gas and nutrient exchange [6]. The EPS represents ca. 90% of the total mass of biofilms and is composed mainly of polysaccharides, enzymes, structural proteins, lipids, biosurfactants, and extracellular DNA [12].

The disruption of microenvironments in biofilms is key to preventing lateral gene transfer and short generation times, since fast microbial evolutionary processes can favor increasingly fit phenotypes, related to bacterial persistence and the acquisition of antibiotic resistance [6,7]. It is important to highlight that the presence of extracellular DNA (eDNA) in the EPS matrix contributes to the cohesion of the structure, serves as a nutrient source, and also facilitates the exchange of genetic information [12]. In this sense, biofilms can even be recognized as incubators of genotypic and phenotypic diversity in the microbial world [4,6].

The biofilms formed along meat processing chains are of special concern, considering the expressive market, with huge producers and exporters of beef and poultry (such as Brazil, the U.S.A., China, and the European Union), which rely on intricate international trade for the distribution of products to diverse regions, impacting food safety and business worldwide [12,13,14].

In this review, we compiled studies on the main foodborne pathogens associated with the resident microbiota in meat producing chains, discussing factors that may lead to their persistence in multispecies biofilms. At the same time, we aimed to analyze recent advances in biofilm control methods applied to the meat industry, including their benefits, limitations, and potential impact on food safety. This is especially important because biofilms are very complex structures that are highly resilient against traditional sanitation methods, which demand the development of innovative solutions, which this review aims to address.

## 2. Methods

This study was a non-systematic review that used mixed methods to collect a combination of qualitative and quantitative data, using convenience sampling. This study was conducted by searching for abstracts and full texts, which were read according to the authors’ interest and experience, followed by the development of topics based on the selected articles. The databases searched included PubMed, Scopus, Science Direct, Web of Science, and Google Scholar, with special attention paid to the references from the last ten years to capture the state-of-the art of the research area. The word “biofilm” was used in combination with other terms such as “control”, “foodborne pathogens”, “green methods”, “microbial ecology”, “microbiome”, and “food industry”.

## 3. The Main Pathogenic Microorganisms That Form Biofilms and Their Importance for the Meat Industry

According to the literature, the assessment of contamination by *Salmonella* spp., *Listeria monocytogenes*, *Staphylococcus aureus*, and *Campylobacter* spp. is a routine industrial practice to ensure the safety of poultry, pork, beef, and meat products, considering their importance and frequent detection in biofilms formed on processing surfaces [15,16,17,18].

For the purpose of this review, we present studies on the occurrence of foodborne pathogens and background microbiota with a focus on those that persist even after hygiene procedures, which possibly indicates tolerance to cleaning and sanitization processes, in addition to biofilm formation. When available, we also obtained data on the microbiomes found in meat processing facilities. The microbiota encompasses all the microorganisms found in a given environment, while the microbiome is defined as the collection of all the microbial genes from an environment. We also present a discussion about the main factors that contribute to persistence, and, whenever possible, data on hygiene indicators (e.g., mesophilic aerobes and Enterobacteriaceae) are considered because they are meaningful to the estimation of the overall contamination levels of facilities, with possible implications for the protection of pathogens in multispecies biofilms [19,20,21].

### 3.1. Salmonella *spp.*

Much has been studied about the characterization and epidemiology of *Salmonella* sp., which is able to adhere to and form biofilms on different surfaces, showing tolerance to sanitizers used in industrial cleaning processes, such as sodium hypochlorite and quaternary ammonium [22,23].

The persistence of *Salmonella* spp. after the cleaning processes of surfaces in meat processing facilities is a global concern. In Belgium, Zeng et al. [24] observed the survival of *Salmonella* on surfaces after cleaning and sanitization protocols, with rates of 10.4% in the plucking area, 1.5% in the evisceration room, and 2.0% in the meat cutting room. Similarly, Obe et al. [25] documented occurrences ranging from 11.1% to 33.3% across equipment utilized in various stages, including evisceration, scalding, bleeding, and deboning, with *Salmonella* detected on conveyor belts of persistently isolated poultry cuts in the United States. In Canada, Boubendir et al. [26] reported *Salmonella* occurrences ranging between 9% and 21% in two poultry slaughterhouses, with *S*. Heidelberg and *S*. Kentucky identified as the most prevalent serovars. Furthermore, *S.* Enteritidis was found to withstand cleaning and disinfection procedures in a Spanish poultry slaughterhouse, leading to cross-contamination between the bleeding, evisceration, and meat product transport stages [27]. In Italy, Lauteri et al. [28] found that 5.5% of pork processing environments were contaminated by this pathogen.

In Brazil, the prevalence of *Salmonella* spp. on surfaces post-sanitization varied from 0.8% to 16.6% in poultry slaughterhouses [16,29,30], as well as in pig processing facilities [31,32]. The most identified serovars for pigs included *S*. Derby, *S*. Typhi, *S*. Typhimurium, *S*. Infantis, and *S*. Panama [32,33], while for poultry, the prevalent serovars comprised *S*. Heidelberg, *S*. Enteritidis, *S*. Ohio, *S*. Agona, *S*. Kentucky, and *S*. Minnesota [29,30]. An investigation spanning 18 months revealed persistent *Salmonella* contamination correlated with biofilm production on food conveyor belts within a poultry slaughterhouse, primarily attributed to the *S*. Heidelberg and *S*. Enteritidis strains [29]. Due to the significant risk of cross-contamination posed by these microorganisms to products, the meat cutting room stands out as the most extensively studied area within swine and poultry slaughtering facilities in Brazil. Surfaces such as food conveyor belts, knives, steel gloves, and cutting boards have been identified as primary points of contamination [16,29,31,32].

Microbial isolates retrieved from the poultry and swine industries have exhibited resistance to various classes of antimicrobials, along with weak to moderate adhesion capacities on polystyrene surfaces and variable tolerances to peracetic acid and quaternary ammonium compounds [30,31,32,33,34]. Wang et al. [35] investigated the intensive sanitation practices and the survival of *Salmonella* in a beef processing plant, indicating that this practice had notable effects on the microbial composition and diversity of the biofilms found in these environments. However, *Salmonella* survived and persisted on floors, in drains, and on the surfaces of equipment and utensils due to the microorganism’s adaptation to the environment, tolerance to the concentrations of the chemicals used, and establishment of a consortium with other environmental microorganisms. *Pseudomonas* was the environmental microorganism that promoted a conditioning film for the installation, maintenance, and survival of *Salmonella* on stainless-steel surfaces used in poultry and beef slaughterhouses [36,37]. When these interactions were evaluated in in vitro studies, environmental microorganisms from poultry processing industries belonging to the genera *Pseudomonas*, *Comamonas*, *Acinetobacter*, *Flavobacterium*, *Janthinobacterium*, and *Aeromonas* were responsible for the increased *Salmonella* biofilm formation in multispecies biofilms when compared to pure-culture cultivation, therefore highlighting the importance of studying the bacterial community that inhabits the same ecological niches as those of pathogenic microorganisms to develop effective control practices [36]. These factors need to be considered when assessing the occurrence of *Salmonella* spp. in meat processing environments in order to find more effective control strategies.

### 3.2. Listeria monocytogenes

*L. monocytogenes* is a widespread foodborne pathogen in the meat processing industry, with the ability to survive sanitization processes, forming biofilms on abiotic surfaces, which contribute to its persistence across various ecological niches [22,38,39]. *L. monocytogenes* has been documented to contaminate industrial surfaces during pig slaughter and processing [38,40,41], in poultry facilities [16,42,43], in cattle operations [44,45], and on surfaces involved in the preparation of ready-to-eat products [40,41,46,47]. These facts make *L. monocytogenes* a key microorganism to be considered in self-control programs implemented by food industries.

In several countries, the contamination by *L. monocytogenes* has been of great concern for the meat industry, especially due to the detection of antimicrobial-resistant strains. Rugna et al. [41], in Italy, evaluated two pork processing industries, revealing that 8.7% of the surface samples tested positive for *L. monocytogenes*. The preparation area for meat cuts emerged as the most contaminated spot, particularly on cutting surfaces, knives, and gloves. Notably, these isolates exhibited significant resistance rates to antibiotics crucial in treating listeriosis cases, including clindamycin (57%), ciprofloxacin (43%), oxacillin (36%), and levofloxacin (35%). Similarly, Shedleur-Bourguignon et al. [48] reported comparable findings in a Canadian pig slaughterhouse, with conveyor belt surfaces identified as the primary source of contamination for the product. Additionally, Jang et al. [49] reported in South Korea that glove surfaces, saws, and drains were implicated as sources of contamination for beef. In the ham processing industry in Spain, *L. monocytogenes* was detected on 9.16% of surfaces [40]. Phylogenetic analysis revealed a high degree of similarity among the isolates, confirming cross-contamination within the plant. The deboning and slicing tables, conveyor belts transporting sliced products, and knives, gloves, and drains in the deboning and slicing rooms were identified as sources of contamination [40]. Furthermore, a study found the high prevalence (19.4%) and persistence of *L. monocytogenes* in ten production facilities of Iberian and Serrano Spanish ham. The bacterium was identified in samples of equipment and finished products during processing and even after cleaning procedures [47].

In Brazil, the prevalence of *L. monocytogenes* in poultry, pork, and cattle meat processing industries and their products varied between 1.9% and 10.6% across different states [34,38,39,42,43,44,46]. Contamination was commonly observed in areas such as the floors, walls, and drains in the evisceration and cutting rooms, as well as on knives, cutting boards, table surfaces, and conveyor belts made of stainless steel, polypropylene, and polystyrene. In vitro studies showed that these isolates exhibited resistance to ampicillin and clindamycin, along with the presence of virulence-encoding genes [42,43,47]. They also demonstrated an adhesion capacity and biofilm formation on polypropylene and stainless-steel surfaces at both 12 °C and 37 °C, as well as tolerance to sanitizers used in the hygiene routines of industries, such as peracetic acid and sodium hypochlorite [39,40,43]. The persistence of *L. monocytogenes* on conveyor belts in poultry meat cut preparation rooms has also been demonstrated [38,42], and in pigs [39]. D’Arrigo et al. [47] revealed that persistent strains resulting from different samplings for more than two years in ham processing industries presented more genetic factors associated with biofilm formation and mechanisms of tolerance to environmental conditions in comparison with non-persistent isolates. These authors [47] also pointed out shelter sites for the development of biofilms, such as grooves on utensil surfaces and drains, which are hotspots for re-contamination. These findings reinforce the need for the constant monitoring and identification of potential ecological niches for the control of this pathogen [47].

### 3.3. Staphylococcus aureus

*S. aureus* is widely distributed in nature, and it can contaminate equipment, utensils, and meat products. Considering it is commonly present on the skin and in the mucous membranes of food handlers, its monitoring is very important for industrial self-control programs [50]. *S. aureus* possesses genetic factors primarily associated with the QS system, regulated by the Agr and LuxS/Autoinducer-2 (AI-2) system [51]. These factors govern the adhesion, biofilm formation, virulence, and motility of the microorganism, thereby facilitating its colonization of abiotic surfaces [50,51].

Gutiérrez et al. [18] conducted a study in Spain revealing the occurrence of 3.2% of *S. aureus* on surfaces such as meat grinders, knives, and plastic curtains following the cleaning and sanitization process. The isolated strains harbored staphylococcal enterotoxin genes along with others associated with biofilm formation (*ica*A, *ica*D, and *bap*). Interestingly, multispecies biofilms comprising *S. aureus* and other bacteria including *Pseudomonas*, *Acinetobacter*, *Psychrobacter*, and *Serratia* were detected in the same samples, suggesting that symbiosis may contribute to increasing biofilm formation and the persistence of the pathogen.

In a cattle slaughterhouse in India, Gowda et al. [52] identified the utensils, cutting surfaces, and conveyor belts used for slaughter, as well as the hands of employees, as the primary sources of pathogen contamination for the products. Moreover, Komódromos et al. [53] reported on a study in cattle, pig, and sheep slaughterhouses in Greece, showing that *S. aureus* was present on animal carcasses, in the mucous membranes of food handlers, and on industrial surfaces. The occurrence of *S. aureus* ranged from 4.2% to 31.7% among the slaughterhouses, and the isolates were able to form biofilms.

In Brazil, despite low counts, *S. aureus* has been detected on knife surfaces, tables, cutting boards, and the hands of employees during pork processing. Of concern, the isolates were categorized as methicillin-resistant (MRSA), posing a potential problem for the Brazilian pork chain [54].

### 3.4. Campylobacter *spp.*

*Campylobacter coli* and *Campylobacter jejuni* are predominant foodborne species that are often part of the intestinal microbiota of broiler chickens, making poultry slaughterhouses important focal points in epidemiological studies. Typically, food becomes contaminated with *Campylobacter* sp. due to failures during the plucking and evisceration processes [55].

The occurrence of *Campylobacter* spp. in poultry slaughterhouses varies worldwide. In China, the prevalence ranged from 75% to 98% [56,57], while in Argentina, it was reported at 31% [58], in Algeria, at 62% [59], in Germany, at 36% [60], and in Ireland, at 53% [61]. In Brazil, the occurrence of *Campylobacter* spp. in chicken carcasses slaughtered under official inspection ranged from 36% to 69% [62,63,64,65,66].

García-Sánchez et al. [67] detected *C. jejuni* in a poultry slaughterhouse regardless of the implemented cleaning and sanitization processes, which indicates the environmental persistence of the pathogen and raises great concern, since even carcasses free of campylobacter can become contaminated at the slaughter line. It has been shown by Gomes et al. [15] that *Campylobacter* tolerates harsh environmental conditions in meat industries in Brazil. These authors found that *C. coli* was able to grow at low temperatures (7 °C and 10 °C), survived in the presence of 7.5% NaCl, and was resistant to acid and oxidative stresses. Additionally, the isolates displayed a high diversity of genes related to virulence and stress adaptation. Corroborating these findings from Brazil, Carvalho et al. [17] demonstrated the ability of *C. jejuni* isolates to adhere to surfaces made of stainless steel, polystyrene, and polyethylene at temperatures of 4 °C, 12 °C, and 25 °C in a poultry slaughter environment. These research papers indicate that it is a challenge to control *Campylobacter* spp. in industrial settings.

## 4. Microbial Ecology in Food Industries and Its Implications for the Persistence of Pathogens

The microbiota from various meat facilities, equipment, and utensils often correlates with food production processes, indicating that these may represent a natural habitat for multispecies biofilms [68]. Moreover, environmental factors can shape the abundance and diversity of microorganisms, including the surface material, the equipment design, organic debris, the quality of the raw materials and water, employee practices, in addition to the kinds of chemicals used for cleaning and sanitation [69]. These factors contribute to the selection of either a resident or transient microbiota, adapted to specific industries or different areas within the same facility. The resident microbes can be established in a particular location when favorable conditions support their adhesion, growth, and colonization. However, a transient microbiota can also inhabit these sites, leading to changes in the composition of the resident microbiota over time, often triggered by the stresses of cleaning and sanitization practices, temperature fluctuations, and microbial competition [68,69].

The contamination by foodborne pathogens in industrial food premises has been largely studied, but research on the diversity of background microbiota has been investigated more in depth only in recent years, with the advent of high-throughput molecular biology techniques (Table 1). The survival of pathogenic microorganisms can be modified by interactions with the resident and/or transient accompanying microbiota, which can impact the control methods, for example, by protecting them from the action of antimicrobials. The mechanisms involved in the protection of microorganisms in biofilms include the delayed penetration of antimicrobial agents into the biofilm matrix, the altered growth rate of sessile organisms, and other physiological changes observed in the biofilm mode of growth [70]. So, knowing the structures and interactions of the microbiotas that persist in the environments of meat processing plants may clarify the factors that are favorable or not for the survival of pathogens or spoilage microorganisms in order to pave the road for proposing efficient methods of controlling undesirable microorganisms.

In Table 1 are shown recent data on microbial communities attached to meat industry surfaces.

In general, Table 1 shows that *Pseudomonas*, *Psychrobacter*, *Bacillus* and *Acinetobacter* (common in soil, water, and vegetation) have been often detected on sanitized surfaces, suggesting that psychrotrophic microbiota carried out by raw materials is very resilient to cleaning and sanitization procedures, with the ability to colonize and thrive in the cold environments of the meat industry [38,74,75,76,77,83,84,85].

More specifically, in the research conducted by Bridier et al. [71] in France, the prevalence and antimicrobial resistance of stainless-steel-surface-associated *Salmonella* isolates were investigated in a pig slaughterhouse, before and after cleaning and disinfection procedures (chlorinated alkaline solution daily along the chain; ethanol-based solution additionally sprayed on cutting blades; acid foaming solution weekly along the slaughter chain). These authors [71] reported that *Salmonella* was isolated from 67% of the samples analyzed (presence or absence), with no significant differences between the numbers of positive samples before and after cleaning and disinfection. Also, they [71] reported that in the short term, those procedures did not modify the profile of the *Salmonella* susceptibility to antimicrobials. In contrast, results from metabarcoding studies (*16S* rRNA) conducted by Bridier et al. [71] with surface-associated samples from a pig slaughterhouse indicated a decrease in the alpha-diversity index after cleaning and disinfection, regardless of the chemicals used. Moreover, these authors [71] showed that the Moraxellaceae family (*Acinetobacter*, *Moraxella*, *Psychrobacter*, and *Enhydrobacter*) dominated the bacterial communities studied, and they observed a clear negative correlation between *Enhydrobacter* and *Salmonella*. According to the literature [86], the increased survival of bacterial pathogens in multispecies biofilms can be attributed to protection by the extracellular matrix or complex interactions among microorganisms. It is interesting to note that Bridier et al. [71] suggested that the *Psychrobacter* genus could be a useful model to study bacterial adaptation in food environments, considering it is dominant after cleaning and disinfection, as determined by metataxonomic studies.

In a pig processing plant in Spain, Hascoët et al. [72] studied *L. monocytogenes* and the resident microbiota from surfaces (stainless steel and polypropylene) after cleaning and disinfection processes. For this, surface sensors were implemented, sampled, and evaluated by culture plate count. The results [72] showed that *Bacillus* spp. was the most prevalent genus, but 57.27% of the aerobic mesophilic bacteria belonged to Gram-negative species, mostly *Pseudomonas* spp., *Mannheimia haemolytica* (an opportunistic respiratory pathogen in animals), and *Rhizobium radiobacter* (a ubiquitous bacterium). Lactobacilli and other LAB were also detected, likely from starter cultures (cured meats) or alternatively from raw material cross-contamination [72]. These authors [72] grouped the microbiota into two clusters: (1) dominated by *Pseudomonas* spp., with samples from successive stages of the productive process; (2) dominated by *Bacillus* spp., from close areas sharing personnel and equipment. Moreover, the results suggested that there was cross-contamination along the processing, since there were bacteria allocated in both clusters [72]. Regarding *L. monocytogenes* contamination [72], it seemed to be associated with *Pseudomonas* spp. and enterobacteria, which are many times considered as primary colonizers with the ability to produce sticky substances, important to protect pathogens in biofilms. The best knowledge of biofilm ecology may contribute to unravelling communities that may displace the pathogen from the meat processing environment.

The resident microbiota from a Spanish porcine slaughterhouse was also studied by Calero et al. [73], but this time shotgun metagenomics was used, indicating that bacteria prevailed in the arrival and anesthesia zones, ranging from *Actinobacteria* to *Firmicutes* and *Proteobacteria*. Interestingly, based on genomic functional analysis, the authors [73] reported that there was a high prevalence of stress-associated SOS-responsive genes in the microbiota in the early zones, which could indicate genome changes important for biofilm formation and antibiotic response, among other traits. Moreover, the authors [73] highlighted that to reduce microbial contamination, it is important to implement good disinfection strategies, mainly using HLE (disinfectant composed of EDTA, lactic acid, and hydrogen peroxide) after washing with detergent.

There was yet another report from Spain by Cobo-Díaz et al. [74] on a newly opened facility that produced packed, fresh pork meat, which was sampled all along the cutting process to search for the non-transient microbial diversity and antimicrobial resistance genes using culture-dependent and culture-independent methods. These authors observed the dominance of *Pseudomonas*, *Psychrobacter*, and *Acinetobacter*, with the latter genus associated with antimicrobial resistance genes related to certain antimicrobials frequently used on regional pig farms. At the same time, they [74] reported a sharp increase in extended-spectrum β-lactamase-producing *Enterobacteriaceae* and vancomycin-resistant *Enterococcaceae* after the start of cutting activities. It was also noteworthy [74] that drains and food contact surfaces were hotspots for antimicrobial resistance genes, with a better resistome detection performance by culture-dependent analyses compared to shotgun metagenomics.

A pork industry from Austria was studied by Zwirzitz et al. [75], collecting samples from multiple sites to determine the environmental-associated microbiota co-occurring with *Listeria* sp. and *Listeria monocytogenes*, based on cultivation and high-throughput full-length *16S* rRNA gene sequencing. Contamination by the *Listeria* genus was widely disseminated (50%), while *L. monocytogenes* was present in 13.6% of the samples [75]. Moreover, there was an association of *Listeria* spp. with the known meat-spoilage biofilm-producing genera *Pseudomonas*, *Acinetobacter*, and *Janthinobacterium*. The exact metabolic interactions were not elucidated, and it was not possible to determine whether listeria drove the shifts in the microbial communities or, on the contrary, pre-existing microorganisms in the biofilms favored listerial survival [75].

A swine slaughterhouse was also studied in Canada by Cherifi et al. (2022) [76], and the samplings were conducted on conveyor belt surfaces during four visits and after the daily cleaning and disinfection procedures, based on quaternary ammonium compounds. The persistent microbiota was determined using the *16S rRNA* gene sequencing method, while the presence or absence of *L. monocytogenes* was checked by the culture enrichment method [76]. Moreover, to explore the relationships, particularly between *Listeria* and other genera, a network of positive and negative correlations was created [76]. The results by Cherifi et al. (2022) [76] revealed that *L. monocytogenes* was cultured from 27.1% of the samples analyzed and, interestingly, the alpha diversity indexes were similar for culture-positive and culture-negative samples. The most abundant genera identified on the conveyor belts [76] were the Gram-negative *Sphingomonas*, *Pseudomonas*, *Acinetobacter*, *Chryseobacterium*, and *Caulobacter*, confirming their importance as residents of surface-associated microbiota in meat plants. At the same time, *Listeria* and *Staphylococcus* were among the most abundant genera in the *Firmicutes* phylum detected by metataxonomics on the cleaned meat conveyor surfaces, leading to the hypothesis that *Listeria* could form niches within *Pseudomonas* biofilm, being protected from harsh conditions [76]. However, these authors [76] also reported that based on community network analyses, only negative correlations between *Listeria* and other genera were detected. For example, *Sphingomonas* was found to interact negatively with *Listeria* spp., likely due to growth inhibition, the absence of shared habitats, or competition, which is interesting in the approach of identifying potential bacterial species to eradicate listeria from the food production environment.

Tadielo et al. [38], evaluating the microbial ecology present on equipment and utensil surfaces in dairy, poultry, and swine slaughterhouses after sanitization in Brazil, indicated that each industry had its own microbial ecology. *Pseudomonas*, *Acinetobacter*, and *Aeromonas* were more prevalent in the poultry slaughterhouse; *Acinetobacter*, *Pseudomonas*, and *Brevundimonas* were more prevalent in the swine slaughterhouses; and *Pseudomonas*, *Kocuria*, and *Staphylococcus* were more prevalent in the dairy industry. Using alpha and beta diversity indicators, the study by Tadielo et al. [38] showed that industries that processed meat products shared the same microbial ecology, demonstrating that the type of product processed also affected the microbial ecology. The high occurrence of *Pseudomonas* in all industries evaluated confirmed its importance as a member of the resident microbiotas of these facilities, and these authors suggested that this primary colonization could possibly favor the persistence of *L. monocytogenes* on different surfaces after sanitization.

Sui et al. [77] evaluated samples from different surfaces before and after sanitation in a swine slaughterhouse in China and reported the high tolerance of *Psychrobacter* and *Acinetobacter* to the process. These microorganisms showed a positive correlation with the occurrence of *Salmonella enterica*, a fact that contributed to the increase in cross-contamination between the products and facilities. Corroborating that study, Jeong et al. [78] highlighted that *Acinetobacter*, *Psychrobacter*, and *Pseudomonas* were the most prevalent genera in clean areas of a poultry slaughterhouse, being associated with the occurrence of *L. monocytogenes*.

The study by Alvarez-Molina et al. [79] demonstrated that *Pseudomonas*, *Psychrobacter*, *Acinetobacter*, *Staphylococcus*, and *Lactococcus* were the most prevalent genera in facilities that processed meat and dairy products in Spain. The analysis of the resistome [79] indicated that these genera were linked to the presence of antimicrobial resistance genes, mainly related to aminoglycosides, tetracyclines, and quaternary ammonium compounds. Although these authors [79] did not explore possible interactions between background microbiota and pathogenic bacteria, their findings reinforced that the spoilage microbiota may be a reservoir for antimicrobial resistance genes [79].

Sequino et al. [80] showed that in the Greek meat processing industry, *Brochothrix*, *Pseudomonas*, and *Psychrobacter* were the most relevant microorganisms, responsible for harboring genes of antimicrobial resistance, biofilm formation, and tolerance to sanitizers, emphasizing the need to understand the microbial ecology to improve the sanitation [77,78,80].

In a study by Maes et al. [81], the contamination of food contact surfaces of seven Belgian food companies was evaluated after cleaning and disinfection procedures, including two meat processing facilities. The microbiotas from the facilities were evaluated by the culture method, and colonies from non-selective culture media were picked up for identification by *16S rRNA* amplicon sequencing. The authors highlighted the spoilage potential of the resident microbiota and reported that *Microbacterium* was the most abundant genus of Gram-positive bacteria, while *Pseudomonas* was the most prevalent Gram-negative bacteria.

Data from surface samples from Spanish meat premises were reported by Rodríguez-López et al. [82] based on the cultivation of *L. monocytogenes* and metagenetic analysis targeting the *16S* rRNA gene. They reported an overall *L. monocytogenes* incidence of 12.54%, and high amounts of *L. monocytogenes*-associated psychrotrophic microbiota were obtained in all cases. Moreover, the metabarcoding analyses revealed that *Actinobacteria* was the phylum most abundant in the meat industry surveyed [82]. Subsequent analysis of the Operational Taxonomic Units showed the co-occurrence of a wide variety of taxa with *L. monocytogenes*, including spoilage-associated genera and lactic acid bacteria, which indicated the ability of the pathogen to thrive in different microbial communities and ecological niches.

Caraballo Guzmán et al. [83] studied food processing plants from Colombia and characterized bacterial communities from biofilms by using *16S* rRNA sequencing by analyzing samples obtained after cleaning and disinfection processes. Four phyla, Proteobacteria, Firmicutes, Actinobacteria, and Bacteroides, represented over 94% of the Operational Taxonomic Units detected, with the two most frequent genera, *Pseudomonas* spp. and *Acinetobacter* spp., representing 93.47%. These authors [83] concluded that different bacterial genera can coexist inside biofilms and persist in production environments, representing a constant risk for manufactured foods.

Wagner et al. [84] assessed the presence of biofilms within an Austrian meat processing environment (pork, poultry, and beef), with the detection of microorganisms (cultivation and targeted quantitative real-time PCR based on 16S rRNA) and at least two biofilm matrix components (carbohydrates, extracellular DNA, and/or proteins). Five biofilm spots were detected on food contact surfaces (cutters, screw conveyor) and five on non-food contact surfaces (drains and water hoses). The most prevalent bacteria in the biofilms belonged to the genera *Brochothrix* (ca. 80%), *Pseudomonas*, and *Psychrobacter* (ca. 70% biofilms), with the presence of multispecies biofilms [84].

Other than the main pathogenic bacteria mentioned in Table 1, it is worth noting that spore-forming microorganisms may be of concern for the food industry. In this regard, *Bacillus cereus* presents the ability to form hydrophobic spores covered with long appendages, which are particularly problematic because they present strong adhesion to inert materials largely used in food industry equipment, with a high tolerance to cleaning and sanitizing procedures [85].

Taken altogether, data about the prevalence of background microbiota and foodborne pathogens after cleaning and disinfection processes in slaughterhouses and meat processing plants [38,71,72,73,74,75,76,77,78,79,80,81,82,83,84] can lead to two main hypotheses: (i) multispecies biofilms may equally offer protection to pathogenic and non-pathogenic bacteria against detergents and antimicrobials by hindering them from the action of chemicals and also serve as incubators of microbial diversity that favor the selection and survival of the fittest; (ii) there is a resident psychrotolerant recalcitrant microbiota that is protected in biofilms and plays a role as a primary settler, paving the road for further colonization by environmental bacteria or those carried out from raw materials by cross-contamination. Either way, it is very important to consider the background microbiota when designing strategies to combat biofilms.

Another key point in studying biofilms in industrial settings is the choice of method to determine the structure of the microbial community, since non-culture-based methods are the best to reveal microbial diversity, but for the detection of pathogens, enrichment-based methods are the best choice, especially considering the availability of isolates to set up models to study multispecies biofilms. Moreover, culture-dependent techniques are still essential for assessing the efficiency of antibiofilm strategies.

## 5. Hidden Places with Potential for Biofilm Formation

As previously reported [38,71,72,73,74,75,76,77,78,79,80,81,82,83,84], the meat processing industry faces persistent challenges with microbial biofilm contamination. These structures are highly resistant to sanitization and difficult to eradicate, posing significant concerns for self-regulation programs and the quality of meat products. These sessile microbial communities successfully tolerate the adverse conditions of cleaning and sanitizing operations, especially due to the protection provided by the exopolysaccharide matrix (the low diffusion rates of sanitizers) and can also be protected by physical barriers (for example, places not accessible for scrubbing) [1], in addition to serving as reservoirs for the constant adhesion of new microbial agents, contributing to microbial diversity (pathogenic and deteriorating microorganisms) and antimicrobial resistance and bacterial stress genes [38,71,72,73,74,75,76,77,78,79,80,81,82,83,84]. In addition, any area or surface whose hygiene is difficult allows food residues and water to be deposited, forming the conditioning films that increase the possibility of microbial adhesion [76,83].

**Figure 3:**
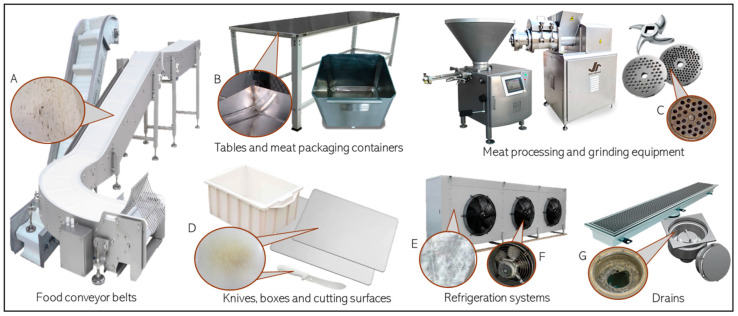
Main hotspots for biofilm formation in meat processing industries. (**A**,**D**) Grooves and cracks in plastic surfaces caused by excessive handling time or friction with bony parts of meat cuts. (**B**) Table edges, prominent welds, and hidden corners. (**C**) Accumulation of organic matter on discs and knives used for cutting meat. (**E**,**F**) Excessive condensation and accumulation of organic material in areas that are difficult to access for cleaning, such as areas near walls (back of equipment) and fan blades. (**G**) Accumulation of waste (meat particles, fat, blood, and other organic material) in drains. Source: the authors.

In the meat processing industry, surfaces that can harbor biofilms are often made from stainless steel, polypropylene, polyurethane, and nylon, as these materials are commonly used in the manufacture of equipment and utensils [87]. In this section, we will briefly discuss the main hotspots that contribute to biofilm formation in the meat industry, focusing on both food contact surfaces (such as conveyor belts, cutting boards, knives, meat grinders, mixers, and boxes) and non-food contact surfaces (including drains, floors, and walls). We will also cover strategies to mitigate the associated risks, as shown in Figure 3.

Equipment and utensils used in meat processing can be sources of contamination due to three main factors: (1) flaws in the industrial sanitary design, such as joints, poorly finished welds, corners, or areas that are difficult to access; (2) excessive wear and occasional maintenance or the late replacement of equipment, often associated with grooves, gaps, and cracks; and (3) the disassembly of equipment during hygiene procedures [87]. Regardless of the cause, these areas serve as sites for the accumulation of meat residues and liquids, creating a favorable environment for microbial adhesion and maintenance, which reduces the effectiveness of control methods [71,84].

*Food conveyor belts and cutting surfaces*: These structures, usually used in meat processing, represent another critical point because they are made of plastic and porous material (polyurethane, polystyrene, and nylon) and are often made up of cracks. These conditions are necessary to maintain mobility within the industrial area, especially in modular conveyor belts [87]. These areas, in addition to enduring the mechanical and abrasive effects of meat cuts (particularly from bones), maintain high humidity and can serve as reservoirs for organic particles (Figure 3A) [38,39,43,84]. Furthermore, operational failures during sanitation, such as inadequate cleaning of the rear part of conveyor belts and their side edges, can significantly increase microbial contamination [38,39]. No less important, even if a surface shows no visible signs of wear, it is well known that, from a microscopic perspective, wear is always present and can be quantified using the roughness index [88,89]. Roughness becomes more pronounced over time and is considered a challenge for cleaning, even though it is not directly associated with the so-called “hidden places”. Chaturongkasumrit et al. [90] evaluated the roughness percentage of polyurethane surfaces and found that new surfaces had low roughness (0.05 ± 0.00 μm), whereas materials used for more than five years exhibited significantly higher roughness (1.44 ± 0.01 μm). The increase in roughness directly impacts contamination by pathogenic microorganisms and reduces the effectiveness of hygiene practices.

*Meat processing and grinding equipment*: Meat grinders and mixers, equipment for obtaining mechanically separated meat (MSM), and other similar equipment are made up of parts with recesses, often small, and which need to be dismantled at the end of each shift because, without this, contamination and colonization will be inevitable (Figure 3C) [91]. It is important to understand that these types of equipment present a high risk of biological contamination (Figure 3C) [92,93]. Especially in relation to MSM, in addition to being a stage with equipment that is difficult to access and sanitize, it also involves working with more contaminated raw materials, providing a greater risk for microbial adhesion [94].

*Deboning the chicken carcass to prepare various cuts of meat*: This procedure is performed using a series of specialized automated machines and overhead rails, consisting of hooks, rods, and irregular surfaces that facilitate the accumulation of organic material during processing, serving as sources of contamination throughout the industrial environment [95]. If equipment and utensils are not properly dismantled, it will be impossible to perform the necessary hygiene procedures to prevent and remove biofilms in these areas [38,78].

*Tables*: Another aspect related to hidden and hard-to-reach areas is the edges of tables, which require close attention regarding biofilm formation. It is common for table finishes to have angles that are difficult to access or that even feature blind spots (Figure 3B) [81,82,83,84,96]. Basically, it is essential not to purchase tables with angled edges. However, if the company already owns them and cannot replace them, an innovative solution is needed to soften the angles or close these blind corners.

*Knives*: Utensils such as knives, despite not representing one of the biggest problems for the formation of biofilms, need to be sanitized as often as necessary to ensure the elimination of contamination (Figure 3D) [81,82].

*Refrigeration systems*: Refrigeration systems and cold storage areas, including condensers and fans, are prone to biofilm formation due to water condensation (Figure 3E,F). Furthermore, these areas do not receive daily operational cleaning, as they primarily serve for the storage of meat products awaiting processing or packaging [74,77,84].

*Drains*: Drains and material flow channels in cutting rooms and other areas of the meat industry accumulate waste during use, including meat particles, fat, blood, and other organic materials from slaughter and processing. However, these areas are often neglected in cleaning and hygiene programs and can be considered hidden spots, as they provide a rich source of nutrients for microorganisms, facilitating the persistence of microbial biofilms (Figure 3G) [84,96,97,98].

## 6. Biofilm Control

An effective cleaning procedure must remove organic debris from surfaces and break down the EPSs from biofilms to expose both the dispersed and sessile cells to antimicrobial agents [98]. The disinfection process aims to decrease the population of cells that remain viable on the surface after cleaning to avoid microbial growth before production is resumed. Nonetheless, the use of chemical agents (e.g., phenols, alcohols, and surfactants) in food processing facilities can damage equipment and lead to the formation of harmful by-products, in addition to increasing the risk of recurrent contamination, for example, when peracetic acid and hydrogen peroxide are used [99,100,101]. It is also important to note that biofilm removal can be greatly impacted by the type of food contact surfaces on which the biofilm rests and also by the microbial species involved in its formation [102]. Due to the complex structure of microbial biofilms and the possibility of selecting sanitizer-tolerant genotypes, there is great interest in novel safe and green methods to control biofilm contamination in food facilities [12,98,101], including the use of enzymes, aromatic plants, bacteriophages, nano-based delivery systems, and other eco-friendly technologies, alone or in combination [103,104,105].

**Figure 4:**
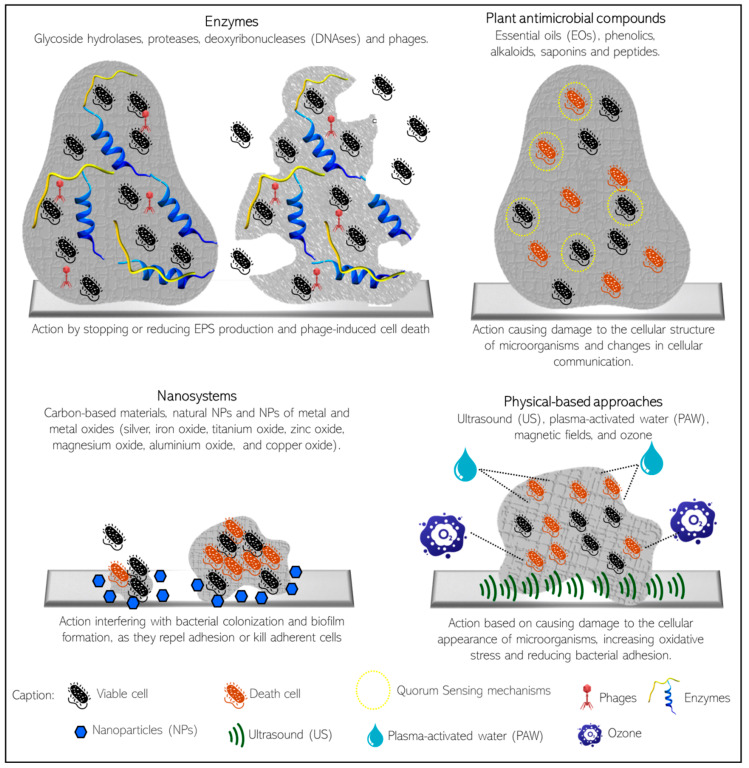
Basic mechanisms of promising technologies for controlling microbial biofilms in industrial environments. Source: the authors.

Taking into account the importance of finding new and efficient methods to control biofilm contamination, especially in the animal-based industry, some of the most promising technologies are illustrated in Figure 4 and are discussed in the following subsections.

### 6.1. Enzymes

Enzymes are considered innovative antibiofilm agents that meet the “green” requirement, as they are biodegradable and non-toxic [106]. In addition, they do not modify the properties of food, being approved by many regulatory agencies for use in combination with detergents as antibiofilm agents for food contact surfaces [106,107,108]. The EPS surrounding the biofilm is usually the main target of biofilm-disrupting enzymes, which can be advantageous over compounds with antimicrobial action since they do not selectively affect bacteria, decreasing the odds of resistance [101,106,109]. Regarding the EPS-disrupting enzymes, there are three major classes: (1) glycoside hydrolases, which cleave glycosidic linkages in the EPS matrix and contribute to the release of planktonic cells from biofilms, which, in turn, are more susceptible to antimicrobials than sessile cells [110,111]; (2) proteases, which cause the hydrolysis of specific peptide bonds between the amino acid residues of proteins and proteinaceous substances present in the EPS, including the cell wall and cell appendages like pili, fimbriae, and flagella, in addition to degrading signaling peptides of intercellular communication of Gram-positive bacteria [99,112,113,114]; and (3) deoxyribonucleases (DNAses), which interfere with the microbial ecology of biofilms, considering that the degradation of eDNA can lead to poor biomass formation, decreased bacterial viability, and higher susceptibility to antimicrobials in young biofilms [114,115,116]. Another enzymatic way to promote biofilm disruption is to use phage-encoded enzymes. Although the intricate structure of the biofilm may make it difficult for phages to access the inner cells of the biofilm, phages can penetrate the EPS matrix by diffusion or with the aid of specific phage-derived lytic enzymes that allow them to actively penetrate the EPS and disrupt biofilms [107,114,115,116]. The literature cites several studies in which phages, or their enzymatic arsenal (peptidoglycan hydrolases, endolysins, and depolymerases), were successfully used to reduce mono- or multispecies biofilms formed by *E. coli*, *E. coli* O157:H7, *S. aureus*, *L. monocytogenes*, and *P. aeruginosa* [99,115,116,117]. More recently, efforts have been directed towards the development of bioengineered phages with improved efficiency in eradicating pre-established biofilms [116,117,118,119,120]. The use of phages to sustainably control unwanted microorganisms in industrial food facilities has advantages over conventional chemical agents, as they target a specific host range and are safe for humans, animals, plants, and the environment, in addition to not causing damage to equipment or surfaces, nor altering food sensorial properties [119]. Although there are a variety of studies and patents that indicate great potential for the use of phage-based approaches for biofilm control in the food industry, there is still a lack of studies on phage resistance mechanisms, the ability of phages to transmit pathogenicity genes, and the traceability of phages in the environment, as well as the detailing of regulatory standards for the use of phages as antibiofilm agents [121].

Quenching of the QS system, named Quorum Quenching (QQ), is also an enzymatic strategy that interferes with different stages of bacterial granule formation by blocking metabolic pathways, and it has been widely discussed in the field of chronic infectious disease control, as it can prevent the growth of drug-resistant strains due to natural selection induced by survival pressure [122,123]. AHL (*N*-acyl homoserine lactone)-based QS is a global regulatory system associated with virulence factors, biofilm formation, and food spoilage [122,123]. AHL (acyl homoserine lactone)-degrading enzymes (lactonases, acylases) naturally produced by microorganisms or chemically synthesized have been successfully studied for biofilm control [124,125,126,127]. It is worth noting that oxidoreductases can also affect QS, as they can modify the chemical structure of AHL rather than degrade it [98,125,128].

Nowadays, there is a wealth of studies in the literature that illustrate the positive antibiofilm effects of enzybiotics (enzyme-based antibacterials), solely or in combination, and some of these studies are presented in Table 2.

Although enzymatic approaches can be quite efficient, it is important to highlight that the heterogeneity of the EPS matrix can substantially limit their action. For instance, Ellis et al. [111] recently performed a study evaluating the ability of 76 diverse recombinant glycoside hydrolases to disperse *S. aureus* biofilms and observed that the ability to degrade biofilms is not inherent to all glycosidases. The study [111] concluded that antibiofilm activity cannot be predicted from activity on pure substrates, which do not represent the reality in environments such as industrial ones. However, when used in combination, or with other agents, EPS inhibitors can significantly improve the antibiofilm activity against different Gram-positive and Gram-negative species [121,137,138,139]. The association of protease type-I (PtI) and α-amylase was significantly more effective at removing *S. aureus* and methicillin-resistant *S. aureus* (MRSA) biofilms than PtI alone [138]. However, Stiefel et al. [140] observed that, although a mixture of proteases completely removed *S. aureus* biofilms from polystyrene surfaces, it was necessary to use a mixture of proteases and polysaccharidases to eliminate *P. aeruginosa* biofilms, which draws attention to the need to know the microorganisms commonly present in biofilms associated with specific environments.

Based on information from the specialized literature, it is undeniable that enzybiotics are valuable allies in combating biofilms. However, this technology still has low commercial availability, mainly due to the high cost when compared to chemical agents and low industrial accessibility, as the technology and production of enzymes and enzyme-based detergents are still expensive and mostly protected by patents [103,105]. Also, there are challenges for the large-scale application of enzymes, such as the establishment of efficient expression systems for their production and the elucidation of the exact mechanisms of action, including the antibiofilm activity [101].

### 6.2. Plant Antimicrobial Compounds

Vegetables naturally produce a myriad of secondary bioactive metabolites, also called phytochemicals, that act as a chemical line of defense against numerous environmental stresses, such as harmful microorganisms [141]. They present multiple mechanisms of antimicrobial activity, for example, the decreased permeability and destabilization of the cytoplasmic membrane, interference with microbial metabolism, and the inhibition of the synthesis of cell walls and nucleic acids. Regarding the antibiofilm effects of phytochemicals, they are mainly related to the suppression of microbial adhesion and attachment, the inhibition of EPS formation, the decreased production of virulence factors, and the blockage of the QS network [103,142,143]. Based on their chemical structures and biological properties, plant antibiofilm compounds are basically grouped into essential oils (EOs), phenolics, alkaloids, saponins, and peptides [103,141].

EOs are complex mixtures of numerous volatile compounds (e.g., aldehydes, phenols, terpenic alcohols), present in a variety of aromatic plants [144,145]. The chemical compositions of EOs vary according to the parts of the plant used (leaves, barks, roots), crop conditions (nutrient availability, climate, seasonality), and extraction methods [146]. EOs associated with antimicrobial properties include *Thymus vulgaris* (thyme), *Cymbopogon flexuosus* (lemon grass), *Cinnamomum zeylanicum* (cinnamon), *Lippia origanoides* (rosemary pepper), *Rosmarinus officinales* (rosemary), *Eucalyptus* sp. (eucalyptus), and *Salvia officinales* (sage), among others [144,145,146]. As several EOs present a Generally Recognized as Safe (GRAS) status, and due to their rich phytochemical diversity, the literature is abundant in studies that evaluate the application of EOs for controlling and eradicating biofilms of undesirable microorganisms in the food industry [146]. Caceres et al. [144] observed that EOs extracted from *L. origanoides* (especially thymol-carvacrol-chemotype II), *T. vulgaris*, and *Cymbopogon martini* (palm rose) showed prominent antibiofilm activity, promoting a 73–75% reduction in biofilm formation from *E. coli* O157:H7, *E. coli* O33, and *Staphylococcus epidermidis.* Interestingly, these authors also observed that the EO with the greatest antibiofilm activity (thymol-carvacrol II from *L. origanoides*) did not affect the growth rate of planktonic bacteria, which prompted the hypothesis that the EOs studied acted by disturbing the QS mechanisms important for biofilm formation.

*Mentha piperita* (peppermint) EO was tested against planktonic cells and biofilms of *V. parahaemolyticus*, *L. monocytogenes*, *P. aeruginosa*, *E. coli* O157:H7, and *S.* Typhimurium, indicating a 3.5- to 6-log decrease in the populations, regardless of the food contact surface tested [147]. The authors also demonstrated that peppermint EO caused the disruption of cell–cell connections, membrane damage followed by the leakage of intracellular components, as well as inhibited genes related to biofilm formation, and it killed both planktonic and sessile cells. Concerning EO isolated bioactive compounds (EOCs), Orhan-Yanıkan et al. [148] evaluated the capacity of four EOCs (carvacrol, eugenol, thymol, and vanillin) with known antimicrobial activity against biofilm-producing *Acinetobacter baumannii* and *E. coli* isolated from a meat industry in Turkey. Although all the EOCs showed antibiofilm properties, the carvacrol and thymol were more efficient, decreasing the cell biomass by ca. 60% for *A. baumannii* and by ca. 63% and 80% for *E. coli* strains, in addition to significantly reducing the viability of the biofilm cells.

Phenolic compounds are also among the main bioactive secondary plant metabolites, and they include phenolic acids, flavonoids, stilbenes, and tannins, as well as their synthetic and semi-synthetic derivatives [142,144]. The inhibitory efficacy of phenolic compounds depends on the microorganisms present in the biofilm, the dose, the exposure time, and the type of polyphenol [144,149]. It has been demonstrated that cinnamaldehyd (a flavonoid present in cinnamon bark extract) presents antimicrobial and antibiofilm activities against a range of Gram-positive and Gram-negative bacteria, such as MRSA, *L. monocytogenes*, *E. coli*, *Vibrio* sp., and *P. aeruginosa* [150,151]. Cinnamaldehyde and its derivative have been shown to inhibit AI-2 mediated QS, decreasing the ability to bind DNA to LuxR, affecting several virulence factors and increasing susceptibility to stress, as well as cytotoxic effects, as verified in cell viability studies [150,151].

Trans-cinnamaldehyde (TC) inhibited and inactivated *Cronobacter sakazakii* on different surfaces, in addition to downregulating genes associated with biofilms [152]. In the study by Olszewska et al. [153], TC showed greater antibiofilm activity against *E. coli* than other plant bioactives (eugenol, citronellol, and terpineol), being able to reduce the microbial metabolic activity by more than 60%, in addition to drastically reducing the cultivability of biofilm cells. Quercetin, a flavonoid abundant in fruits and vegetables, presented antibiofilm properties towards *E. faecalis*, *S. aureus*, enteroaggregative *E. coli*, *E. coli* O157:H7, and *P. aeruginosa* by several mechanisms, such as the inhibition and prevention of cell adhesion, the suppression of AHL-mediated QS, the disruption or alteration of the cell membrane, the inhibition of efflux pumps, and the blockade of nucleic acid synthesis [143,144,154,155]. Likewise, some esters of gallic acid (tannins) presented the ability to prevent biofilm development and eradicate mature bacterial biofilms through various pathways, such as the suppression of EPS matrix synthesis, the inhibition of QS signaling, and changes in the plasma membrane permeability [149,156,157].

The flavonoid extract of *Curcuma aromatica* showed antibiofilm activity against *S. aureus*, while the alkaloid extract of the same plant species exerted significant antibiofilm activity against *Bacillus subtillis*, in addition to promoting a high percentage of dispersion (89.22 ± 3.42%) of its bacterial biofilm [158]. Sinomenine, an alkaloid, has been described to have antibiofilm activity against *S. aureus* by blocking the expression of biofilm-related genes (the upregulation of *agrA* and downregulation of *icaA*), which interfere with biofilm formation and dispersion but do not interfere with bacterial growth, exerting no selective pressure for resistance [159].

Saponins are low-molecular-weight bioactive plant compounds with the ability to form stable soap-like foams in aqueous solution (surfactant properties) [160]. Zhu et al. [161] modified the structure of the saponin sapogenin from *Camellia oleifera* and observed that the promoted acylation increased the antibacterial and antibiofilm activities of the sapogenin by about 300 times. Furthermore, they observed that the S-16 sapogenin derivative inhibited *S. aureus* biofilm synthesis by inhibiting the activity of bacterial M1PDH, a key enzyme in *S. aureus* mannitol metabolism, thereby inhibiting the formation of bacterial extracellular polysaccharides. It was also observed that *Camellia* saponins can interfere with and impair biofilm formation by *B. cereus* through different mechanisms, such as the inhibition of the initial adhesion and metabolism of individual bacteria within the biofilm, the downregulation of genes related to biofilm formation, the inhibition of quorum sensing, and a reduction in the secretion of exopolysaccharides and eDNA [160].

In sum, the literature shows a diversity of phytochemical compounds that may be promising scaffolds for the development of new antimicrobials with targeted activity against biofilms without imposing bacterial selective pressure, encouraging their use through the food chain to minimize the risks associated with the use of traditional disinfectants and synthetic antimicrobials. However, one drawback for the wide use of plant antimicrobial compounds as antibiofilm strategies is the difficulty in determining their specific mechanisms of action to target their specific applications against pathogens and/or spoilage microorganisms. Furthermore, despite the GRAS status of several plant compounds, it cannot be ignored that some phytochemicals may have adverse effects (e.g., skin irritation caused by thymol and carvacrol), although these effects have been associated with prolonged exposure and high concentrations of the compounds [162].

### 6.3. Nanosystems

In recent years, the study of nanotechnology-based antibiofilm approaches has gained visibility [94,96]. Nanoparticles (NPs), up to 100 nm in size, have singular attributes, such as high versatility and high surface-to-volume ratios, as well as unique chemical and physical properties, such as lower toxicity and sterilization tolerance, compared to many of their macroscale counterparts, in addition to activity against several microorganisms [162,163]. The use of NP systems can improve the bioavailability, selectivity, and stability of antimicrobial compounds, in addition to reducing their toxicity [163,164]. According to Ikuma et al. [165], NP–biofilm interactions involve the transport of NPs to the vicinity of the biofilm, the initial anchoring of NPs onto the biofilm surface, and the migration of NPs into deeper areas of the biofilm. Interestingly, the antimicrobial properties of some NPs are mediated by direct contact with the cell wall, without the need for NP penetration into the bacterial cell, which reduces resistance concerns and increases the prominence of the use of NPs as biocontrol agents [107,163].

The nature of the interactions between NPs and biofilm matrices is complex and dependent on several factors, such as the NP characteristics, physicochemical and biological composition of the biofilm matrix, and environmental conditions (e.g., pH, temperature, flow conditions) [165]. Different types of nanomaterials have been studied based on their antimicrobial properties, such as carbon-based materials (fullerenes and carbon nanotubes), dendrimers with internal cavities for other molecules, nanocomposites, natural NPs, and NPs of metal and metal oxides, such as silver (Ag), iron oxide (Fe_3_O_4_), titanium oxide (TiO_2_), zinc oxide (ZnO), magnesium oxide (MgO), aluminum oxide (Al_2_O_3_), and copper oxide (CuO) [107,153,163,165,166].

Metal NPs actively interfere with bacterial colonization and biofilm formation, as they can repel adhesion or kill adherent cells [167]. NPs’ photocatalytic properties, under UV and light, also contribute to the destruction of microbial cells due to the generation of Reactive Oxygen Species (ROS) and hydrogen peroxide on the particle surface [168,169,170]. Hamida et al. [171] demonstrated that biogenic Ag NPs had potent antibacterial, antibiofilm, and anti-virulence activities against several pathogenic bacterial strains, including MRSA. The authors [171] observed that MRSA treated with Ag NPs presented morphological changes such as the formation of apoptotic bodies and cell wall injuries. Ag NP activity is associated with plasma membrane disruption and the shedding of intracellular material, the production of ROS, the downregulation of genes associated with biofilm formation, such as the *flu* gene, in addition to the liberation of biocidal Ag+ [170,171,172]. ZnO NPs inhibited the formation of biofilms of foodborne pathogens (*S. aureus*, *S. enterica*, *E coli*) in a dose-dependent manner [173]. Similarly, the dose-dependent antibiofilm effect has been demonstrated for CuO NPs against MRSA and *E coli*, and for MgO and Al_2_O_3_ NPs on planktonic and biofilm forms of antibiotic-resistant *E. coli*, *K. pneumoniae*, and *S. aureus* [170,174,175].

In addition to their employment as antibiofilm agents, NPs are also very attractive as delivery systems for both hydrophobic and hydrophilic antimicrobial compounds, as they can facilitate antimicrobials to directly interact with the complex structure of biofilms, as well as remain active during the different stages of biofilm development; thus, NPs have been ostensibly investigated as antimicrobial delivery systems for controlling biofilms in clinical practice [162,164,176]. Fulaz et al. [177] observed that the administration of vancomycin (VAN) coated with positively charged mesoporous silica nanoparticles (VAN-loaded MSNs) was correlated with the reduced viability of both methicillin-sensitive *S. aureus* and MRSA biofilm cells, which allowed the administration of low concentrations of MSNs while maintaining a high local concentration of the antibiotic around the bacterial cells. Amphotericin B (AmB) coated with trimethyl chitosan (TMC) NPs (TMC-NPs/AmB) was significantly more efficient at inhibiting *Candida albicans* biofilms than AmB alone [178]. Tran et al. [179] developed quercetin (Que) and chitosan (Chi) NPs and observed that the complex Que-Chi NP was more effective than Que alone at suppressing the QS-regulated swimming motility and biofilm formation of *P. aeruginosa*. The authors assigned this performance to the better kinetic solubility of the Que in the complex and the greater exposure of bacterial cells to the bioactive compound, in addition to the synergistic inhibition of QS attributed to its Chi fraction [179]. Regarding food-relevant microbes, ε-poly-L-lysine (PL), a broad-spectrum antimicrobial coated with Chi NPs (ChiNP-PL), presented the strong potential to prevent the formation of or inactivate preformed biofilms of monocultures or mixed cultures of five pathogens, including *L. monocytogenes*, *S. aureus*, *S*. Enteritidis, *E. coli* O157:H7, and *P. aeruginosa* [163].

Edible NPs can also be applied to food equipment to generate coatings that can effectively control the growth of bacteria during food processing, since nanocomposites can decrease bacterial adhesion [107,164,169]. Yoon et al. [180] compared the adhesion of *E. coli* to superhydrophobic (carbon nanotubes–polytetrafluoroethylene—CNT-PTFE) and superhydrophilic (TiO_2_) nanocomposite surfaces under different fluid flow conditions. The authors observed a significant reduction in the bacterial adhesion to both surfaces under the flow conditions analyzed, indicating that nanocoated surfaces can promisingly reduce the risk of cross-contamination between liquid foods and biofilms. Shankar et al. [181] demonstrated that gelatine/ZnO NP nanocomposite films exhibited strong activity against *L. monocytogenes* and *E. coli*, whereas that gelatin film alone had no antimicrobial activity.

As observed in other areas, such as health and agriculture, nanotechnology has the immense potential to provide effective systems for delivering antimicrobial agents, favoring the control of biofilms in food processing environments. However, the chemical synthesis of NPs may present toxicity issues, since hazardous compounds may remain in the NPs in trace amounts that may cause undesirable effects on the health of consumers or the environment. Thus, more research is needed to identify the disadvantages of these processes (for example, the evaluation of the consequences of transferring NPs to food regarding their metabolism, distribution, and safety). One way to alleviate this problem would be to apply “green synthesis” technologies to produce NPs.

### 6.4. Physical-Based Approaches

Ultrasound (US) is an environmentally friendly, physical-based technique that refers to acoustic waves with frequencies greater than 20 kHz, beyond the threshold of human hearing [182]. The effects of US or sonic treatment for controlling microorganisms have been reported since the 1950s, but only in recent decades has this technology been studied by the food industry for microbial control, aiming to improve product quality and safety [168]. Effects associated with US include the formation of small vapor bubbles (cavities) generated due to pressure changes [183]. The application of low-intensity ultrasonic waves can produce stable cavitation and cause reparable damage to cells, as it will not cause the violent collapse of bubbles, while the application of high-intensity waves can lead to irreparable cellular injury, which can culminate in microbial inactivation resulting from cell wall harm, the oxidation of intracellular amino acids, and DNA damage [182,183,184]. Studies on the application of US for the control/prevention of biofilm formation indicate that sound waves influence the process in a frequency-dependent manner. Murphy et al. [185] explored the use of low-frequency, low-amplitude acoustic waves in the formation of biofilms by *S. aureus* and *P. aeruginosa*. These authors [185] observed that the exposition of both pathogens to sound waves resulted in a significant increase in biofilm formation, with the greatest level of biofilm being formed following 48 h of exposure at 1600 Hz for *S. aureus* and 800 Hz vibration for *P. aeruginosa*. In contrast, employing higher-intensity acoustic waves, Lee et al. [186] observed reductions of 7.4% and 19.1% in the biofilm formation by *L. monocytogenes* at room temperature after exposure to 37 kHz ultrasonic waves for 5 and 100 min, respectively. Similarly, Charoux et al. [187] employed an airborne acoustic ultrasonic system at a frequency of 26 kHz for 15 min to treat *E. coli* biofilms formed on stainless-steel surfaces and observed significant reductions in the number of adherent cells (between 1.17 ± 0.24 and 2.19 ± 1.01 log10 CFU/mL). When ultrasonic treatment was associated with plasma-activated water (PAW), the antibiofilm effect was even more pronounced, reducing the adherent cell count by 2.2–2.62 log10 CFU/mL when compared to the control treated with distilled water [187].

PAW refers to water that has been treated by a stream of ionized gas (plasma), which is rich in ROS and reactive nitrogen species (RON) [188]. Other plasma-activated liquids (PALs) include sodium chloride, sodium hypochlorite, buffers, and culture media. The use of PAL for microbial control by the food industry is interesting because it is ecological, does not leave residues after treatment, and minimally interferes with the sensory properties of food [189]. PALs can be generated using different techniques, and the inactivation of microbes is influenced by the type of plasma discharge used [189]. Numerous studies report the antimicrobial activity of PALs against planktonic and adherent microorganisms of interest in food, both pathogens and spoilage agents [188,189,190]. For instance, PAW generated from tap water using a surface dielectric barrier discharge at different discharge powers (26 and 36 W) and activation times (5 and 30 min) was tested by Fernandez-Goméz et al. [191] against biofilms formed on stainless-steel and polyethylene surfaces by a cocktail of three *L. monocytogenes* strains. The authors observed that the PAW generated at 36 W–30 min was the most efficient, resulting in reductions greater than 4 log in both the free and adherent states of *L. monocytogenes* [191]. In the same study [191], RNA-seq analysis identified that the main transcriptomic alterations were related to carbon metabolism, virulence, and general stress response genes, with the strong upregulation of the cobalamin-dependent gene cluster. The combination of PAL and other antimicrobial treatments may also lead to more interesting synergistic effects than the use of the treatments alone. Charoux et al. [187] observed that *E. coli* biofilms formed on stainless-steel surfaces treated with PAW, generated from thermal and cold plasma jets, did not show any significant reductions in the counts compared to the control biofilm treated with distilled water, possibly due to the fact that the studied strain was a poor biofilm former. However, the combined treatment of cold plasma-generated PAW with US caused a significant reduction (approx. 2.2 ± 0.59 log10 CFU/mL) in the biofilm, while *E. coli* biofilm cells were reduced to undetectable levels, resulting from the combination of US and thermally generated PAW.

Magnetic fields (MFs) are physical non-thermal treatments used to control microorganisms in the food industry. While high-intensity pulsed MFs are employed as a sterilization technology during food processing, weak MFs can activate and promote microbial growth [192]. Furthermore, MFs do not alter the sensory characteristics of products, as occurs with thermal sterilization, and they are non-toxic, ecological, and safe. Regarding antibiofilm activity, MFs can alter the permeability of the biofilm to ions and affect the metabolism, biochemical processes, and membrane potential of microorganisms [193]. The performance of MFs in microbial control is dependent on several factors, such as the intensity, exposure time, shape and orientation of the magnets, and type of microbe involved [192,194,195]. Aoyama et al. [195] noticed that *E. coli* biofilm was suppressed on glass surfaces by the 20 kHz electromagnetic field but not by the 30 kHz field, whereas the biofilm formed by *Staphylococcus epidermidis* was suppressed with both tested MFs. More recently, MFs combined with magnetic materials, such as magneto NPs, are considered cutting-edge technologies for biofilm control. External MFs can force magnetic NPs to penetrate the cell wall, resulting in microbial death [196]. Li et al. [193] observed that the combination of magnetic iron oxide NPs and MFs caused significant mechanical disruption to the biofilm matrix, leading to the significant dispersal of MRSA, achieving up to a nearly 5-log10 reduction in biofilm bacteria.

The application of ozone treatment, in aqueous or gaseous form, is another promising antibiofilm technique for food industries, with low environmental impact, as it does not leave harmful residues on food products or food contact surfaces [197]. The antimicrobial activity of ozone is linked to its high oxidative potential, in addition to its reactivity and instability [196]. Both the gaseous and aqueous forms of ozone are approved by the Food and Drug Administration (FDA) for direct contact with different food matrices, including fish, meat, and poultry [198]. However, since exposure to ozone is toxic to humans, there are exposure limits in many countries to protect operators [198]. Marino et al. [199] evaluated the effect of aqueous and gaseous ozone against biofilms of *Pseudomonas fluorescens*, *S. aureus*, and *L. monocytogenes* formed on a stainless-steel surface. The authors observed that, after exposure for 20 min under static conditions, the reduction in the microbial viability ranged from 1.61 to 2.14 for the three microorganisms. Biofilms treated under an ozonated water flow showed reductions in viability between 3.26 and 5.23, with *S. aureus* being the most sensitive species under these conditions. Furthermore, the application of low concentrations (up to 0.2 ppm) of gaseous ozone for 60 min caused a reduction of more than two logarithmic cycles of the tested microorganisms, while higher concentrations were able to reduce *L. monocytogenes* biofilms below the detection limits of the methods, in addition to reducing >4.7 log of *S. aureus* and >5.5 log of *P. fluorescens* biofilms [185]. Based on these results, it is possible to see the usefulness of ozone application in different industrial sanitation protocols. However, despite its environmentally friendly appeal, ozone can cause undesirable sensory changes in foods, especially those containing high lipid contents [200].

### 6.5. Antibiofilm-Coated Surfaces

The kinetics of bacterial adhesion is influenced by the chemical and physical characteristics of the different types of surfaces found in food processing environments, such as the roughness and electrical charges [201]. By hindering microbial growth on surfaces, antibiofilm coatings reduce the risk of cross-contamination between food items and food contact surfaces, improving the safety of the food preparation and handling environment [202].

Coverings can be designed to contain immobilized or non-immobilized antimicrobials [98,107,165,203]. In the first case, antimicrobial activity results from the diffusion of biocides out of the coating (contact-based antimicrobial coating), while in the second example, microorganisms are killed upon contact with the surface (released-based surfaces) [165]. It is also possible to cause changes in the equipment surface topology to prevent or limit bacterial adhesion [98,108,165,203].

Antibacterial surface coatings are loaded with active natural or synthetic antimicrobials that are toxic to microorganisms, destroying or inhibiting their attachment to surfaces, preventing their proliferation, and limiting biofilm formation [202,204]. Valliammai et al. [205] developed a polymeric antibiofilm coating containing citral (CIT) and thymol (THY) for application on titanium surfaces by the spin-coating process and observed that the active biomolecules (CIT and THY) were released in a sustained manner for 60 days. The researchers obtained a uniform coating, as observed by atomic force microscopy, and a reduced surface roughness and coating thickness, as measured by a surface profilometer. Furthermore, the antibiofilm coating successfully prevented MRSA adhesion under laboratory conditions, and the antibiofilm properties of the coating were not influenced by plasma conditioning [205]. Another prolific innovation in the development of antimicrobial surfaces is the application of coated NPs, as previously discussed in Section 6.3 (Nanosystems). Coatings with different types of NPs, such as silver, copper, and antioxidant NPs, have been studied to prevent biofilm formation. However, a drawback of this approach is that the surface erodes and therefore becomes available for biofilm formation [98]. Furthermore, it is worth noting that, in the case of released-based surfaces, antimicrobials are incorporated into the system in a fixed quantity so that the coatings become ineffective when the agents are fully released [165].

Contact-based antimicrobial polymer coatings can also effectively prevent microbial colonization. Different molecules, such as antimicrobial peptides (AMPs), chloropolymers, photocatalytically active semiconductors, and cationic polymers, can be covalently attached to surfaces, inducing bacterial cell death or reducing bacterial metabolic activity [165,206,207,208]. Marjhi et al. [206] immobilized an AMP (KLR) on stainless-steel surfaces via chemical coupling (EDC/NHS). The authors observed that the KLR-modified surfaces resulted in the effective inhibition of both *E. coli* and *S. aureus* by nearly 100% and 95%, respectively [207]. Stable protective films formed by cationic polymers, such as polyethyleneimine (PEI), allow robust adhesion to the substrate, enabling the creation of protective layers that seal surface flaws and protect against corrosion, in addition to presenting biocidal properties linked to the disruption of biomembranes [204,208]. Yushina et al. [208] tested the activity of seven cationic-containing amino group polymers against *L. monocytogenes* and *P. aeruginosa*, and they observed a significant biocidal effect (>95% for both pathogens) in both aqueous solution and after the formation of polymer films on the hydrophilic glass plates. *N*-halamines is a contact-activated antimicrobial chloropolymer that releases oxidative halide groups, such as hypochlorous acid, from its structure [207]. When *N*-halamines encounter bacterial membranes, they transfer their oxidative chlorine groups to the cells, thereby causing their death. Nautiyal et al. [207] tested the antimicrobial activity of polypyrrole (PPy), a nitrogen-based conductive polymer, transformed into *N*-halamine after treatment with chlorine bleach. The PPy *N*-halamine-coated tape film inactivated more than 6-log CFUs of *S. aureus* and *E. coli* O157:H7 within 1 min of contact time.

Functionalizing a surface through the adsorption of a polymer and/or altering the micro- and nanostructure of the surface are strategies designed to hinder or substantially reduce the likelihood of bacteria adhering, thereby preventing the development of biofilms, with the advantage of not creating selective pressure on microorganisms [165,209]. The development of anti-adhesive surfaces includes the use of passive polymers, hydrogels, and poly-zwitterionic polymers, while modifying the surface topography changes the surface properties by creating a hydrophobic or superhydrophobic interface [210]. DeFlorio et al. [211] developed a durable, superhydrophobic coating on stainless steel which resists fouling by *E. coli* and *Listeria innocua*. Superhydrophobic surfaces, such as poly(ethylene glycol) (PEG) and PEG-like hydrophilic polymeric coatings, take advantage of the micro- and nano-scale surface roughness or porosity, combined with low-surface-energy coatings, to create air pockets that prevent bacterial contact and adhesion [98,203]. Studies such as those cited, which favor the understanding of surface adhesion mechanisms or how dead bacteria adhered to the surface can be removed from these surfaces, represent an important advance in the development of new antibiofilm strategies.

Taken all together, it has been well established through numerous studies described in the specialized literature that the presence/persistence of biofilms on surfaces and equipment in food facilities is a key factor that serves as a long-lasting source of contamination that puts food quality and safety at risk. The presence of undesirable biofilms in food processing facilities leaves both industry and academia in a continuous state of alert, and the development/application of new technologies, especially “green” technologies, is necessary to overcome this problem. In addition to the biofilm control methods discussed here, there are numerous others, each with their own advantages and disadvantages. Therefore, their usefulness in cleaning protocols for food processing environments must take into account the microorganisms most likely to be in the environment forming biofilms, the kinds of surfaces, as well as the type of food and the safety of the method. Furthermore, it is essential to consider that the synergistic effects of combining antibiofilm methods are likely to be more effective than using isolated approaches to ensure consumer safety.

It is also important to highlight that this review did not intend to be systematic and it provides the point of view of the authors about the topic, based on a critical literature review of recent papers on multispecies microbial biofilms important for food (meat) quality and safety, with a special focus on the new insights provided by the advancement of high-throughput DNA sequencing techniques and the novel approaches to controlling biofilms in the industry.

## 7. Conclusions and Perspectives

Biofilms are complex structures that may protect microorganisms from stresses commonly found in industrial environments (e.g., cold, heat, drying, starvation, sanitizers). Moreover, the EPS matrix offers unique spatial and psycho-chemical conditions that favor the survival of complex microbial communities, which have been more recently described thanks to the advent of massive DNA sequencing technologies. These findings have indicated the presence of a persistent background microbiota (e.g., *Pseudomonas*, *Psychrobacter*, and *Acinetobacter*) even after regular cleaning and sanitation processes carried out in the meat industry. In this way, not only classic foodborne pathogens should be addressed in self-control programs but persistent environmental microorganisms must also be monitored as indicators of biofilm formation.

Moreover, to propose novel strategies for controlling microbial biofilms, research needs to advance to elucidate the mechanisms of bacterial resistance to antimicrobials, as well as to understand the arsenal that helps microbial cells to survive under harsh conditions.

From the point of view of the food industry, it is helpful to implement advanced biofilm detection technologies in order to take action in the early stages to prevent biofilm formation, using eco-friendly antimicrobial strategies whenever possible to protect equipment, consumers, and the environment.

## Figures and Tables

**Table 1:** Description of the microbiota of environmental surfaces, equipment, and utensils from slaughtering and processing industries of pork, beef, and poultry *.

Industry	Country	Type of Surface	Background Microbiota	Other Findings	Reference
Pork industry	France	Stainless steel	*Acinetobacter*, *Moraxella*, *Psychrobacter*, *Enhydrobacter*, *Brevundimonas*, *Rothia*	Positive correlation of *Salmonella* with *Acinetobacter*. Negative correlation of *Salmonella* with *Enhydrobacter.*	[71]
	Spain	Stainless steel, polypropylene	*Bacillus*, *Pseudomonas*, enterobacteria, *Mannheimia*, *Rhizobium*, *Staphylococcus*, *Aeromonas*	Positive correlation of *L. monocytogenes* with *Pseudomonas.*	[72]
	Spain	Stainless steel, polypropylene	*Lactobacillus*, *Acinetobacter*, *Aerococcus*, *Staphylococcus*, *Macrococcus*	Presence of heat shock genes, cell death, biofilm formation, and antibiotic resistance.	[73]
	Spain	Stainless steel, polypropylene	*Pseudomonas*, *Acinetobacter*, *Psychrobacter*	*Acinetobacter* and *Pseudomonas* were reservoirs of resistance genes to β-lactams, tetracycline, and aminoglycosides	[74]
	Austria	Stainless steel, polyurethane polypropylene	*Pseudomonas*, *Psychrobacter*, *Acinetobacter*, *Chryseobacterium*, *Anaerobacillus*, *Anoxybacillus*	Positive correlation of *L. monocytogenes* with *Acinetobacter*, *Janthinobacterium*, *Brachybacterium*, and *Carnobacterium*.	[75]
	Canada	Polypropylene	*Pseudomonas*, *Acinetobacter*, *Sphingomonas*, *Chryseobacterium*	*Pseudomonas* showed a positive correlation with the presence of *L. monocytogenes*. *Sphingomonas*, *Herminiimonas*, and *Bryobacter* were inhibitory towards *L. monocytogenes*.	[76]
	Brazil	Polypropylene	*Acinetobacter*, *Pseudomonas*, *Brevundimonas.*	High occurrence of *Pseudomonas* and *Acinetobacter* in samples after cleaning and sanitizing.	[38]
	China	Stainless steel, polypropylene	*Psychrobacter*, *Acinetobacter*	Samples collected before and after cleaning and sanitizing.	[77]
Poultry industry	Brazil	Polypropylene	*Pseudomonas*, *Acinetobacter*, *Aeromonas*	High occurrence of *Pseudomonas* and *Acinetobacter* in samples after cleaning and sanitizing.	[38]
	South Korea	Stainless steel, polypropylene	*Acinetobacter*, *Psychrobacter*, *Pseudomonas*	Not applicable.	[78]
Beef industry	Spain	Stainless steel, polypropylene	*Pseudomonas*, *Psychrobacter*, *Acinetobacter*, *Staphylococcus*	Genes for resistance to aminoglycosides, tetracyclines, and quaternary ammonium compounds.	[79]
	Greece	Stainless steel, polypropylene	*Brochothrix*, *Carnobacterium*, *Pseudomonas*, *Psychrobacter*	Resistance genes to aminoglycosides, beta-lactams, macrolides, lincosamides, and tetracyclines. Adhesion and biofilm formation genes.	[80]
Simultaneous processing industry of beef, pork, and poultry	Belgium	Stainless steel, polyurethane, polypropylene	*Stenotrophomonas*, *Pseudomonas*, *Microbacterium*, *Rothia*	Isolates showed the production of lipolytic and proteolytic enzymes.	[81]
	Spain	Stainless steel, polypropylene	*Lactobacillus*, *Psychrobacter*, *Microbacterium*	Positive samples for *L. monocytogenes*.	[82]
	Colombia	Stainless steel, polypropylene	*Pseudomonas*, *Acinetobacter*, *Leuconostoc*, *Lactobacillus*, *Streptococcus*, *Escherichia.*	-	[83]
	Austria	Stainless steel, polypropylene	*Brochothrix*, *Pseudomonas*, *Psychrobacter*	Biofilm samples after cleaning and sanitizing.	[84]

* The search for articles was carried out on *Science Direct*, *PubMed*, and *Google Scholar* with the keywords “Microbial ecology, microbiome, and food industry”. Articles published between 2019 and 2024 were selected.

**Table 2:** Applications of antibiofilm enzymes against microorganisms of interest in the food industry.

Enzyme Action Mode	Enzyme Applied	Target Food-Related Biofilm Producer	Surface Material	Reference
Glycoside hydrolases	Diverse recombinant glycoside hydrolases	*Staphylococcus aureus*	Polystyrene	[111]
	CAase	*Escherichia coli* O157:H7, *Salmonella* Typhimurium, *Listeria monocytogenes*	Polystyrene	[108]
	Cellulase	*E. coli* O157:H7	Polystyrene	[129]
	PslG_PF_	*P. aeruginosa*, *Pseudomonas* spp.	Glass	[130]
	α-amylase	*P. aeruginosa*, *S. aureus*	Polystyrene	[131]
Proteases	Serine protease	*Acinetobacter baumannii*, *S. aureus*	Polystyrene	[112]
	Serine protease	*Bacillus cereus*	Polystyrene, Stainless Steel	[113]
	Lysostaphin	*S. aureus*	Glass	[132]
	Serrapeptase	*S. aureus*, * MRSA	Glass	[133]
DNAse	DNase I	*S. aureus*, *P. aeruginosa*	Polystyrene	[134]
Phage encoded enzymes	Endolysin LysH5	*S. aureus*	Polystyrene	[116]
	Endolysin LysCSA13	*S. aureus*	Polystyrene, Glass, Stainless Steel	[135]
	Phage DW-EC (several enzymes)	** EHEC, EPEC, ETEC	Polystyrene, Stainless Steel	[136]
Anti-QS enzymes	LrsL (AHL-lactonase)	*P. aeruginosa*	Polystyrene	[128]
	AiiA (AHL-lactonase)	*Vibrio parahaemolyticus*	Glass	[126]
	AidC (AHL-lactonase)	*P. aeruginosa*	Glass	[127]
Mix of enzymes	DNAse I + Lysostaphin	*S. aureus*	Glass	[133]
	DNase I + proteinase K + cellulase	*E. coli* O157:H7	Polystyrene	[129]
	Protease type-I (PtI) + α-amylase	*S. aureus*, * MRSA, *E. coli*	Polystyrene	[137]
	Protease + lipase + amylase	*S.* Typhimurium, *Cronobacter sakazakii*	Polystyrene, Stainless Steel	[138]
	Auresin + AuresinePlus(proteases)	*S. aureus*	Stainless Steel, Glass	[139]

* MRSA: methicillin-resistant *S. aureus;* ** pathogenic *E. coli:* enterohemorrhagic *E. coli* (EHEC), enteropathogenic *E. coli* (EPEC), and enterotoxigenic *E. coli* (ETEC). The search for articles was carried out on *Science Direct*, *PubMed*, and *Google Scholar* with the keywords “enzymes” and “biofilm control”. Articles published between 2017 and 2025 were selected.

## Data Availability

No new data were created or analyzed in this study. Data sharing is not applicable to this article.
